# CD4 and CD8 T lymphocyte interplay in controlling tumor growth

**DOI:** 10.1007/s00018-017-2686-7

**Published:** 2017-10-14

**Authors:** Dmitrij Ostroumov, Nora Fekete-Drimusz, Michael Saborowski, Florian Kühnel, Norman Woller

**Affiliations:** Clinic for Gastroenterology, Hepatology, and Endocrinology, Hannover Medical School, Carl Neuberg Str. 1, 30625 Hannover, Germany

**Keywords:** Crosstalk, Immunotherapy, Immune escape, T cell exhaustion, Neoantigen, Oncolytic virotherapy, Cancer vaccine, Immune response, Clinical study, Cancer mouse model

## Abstract

The outstanding clinical success of immune checkpoint blockade has revived the interest in underlying mechanisms of the immune system that are capable of eliminating tumors even in advanced stages. In this scenario, CD4 and CD8 T cell responses are part of the cancer immune cycle and both populations significantly influence the clinical outcome. In general, the immune system has evolved several mechanisms to protect the host against cancer. Each of them has to be undermined or evaded during cancer development to enable tumor outgrowth. In this review, we give an overview of T lymphocyte-driven control of tumor growth and discuss the involved tumor-suppressive mechanisms of the immune system, such as senescence surveillance, cancer immunosurveillance, and cancer immunoediting with respect to recent clinical developments of immunotherapies. The main focus is on the currently existing knowledge about the CD4 and CD8 T lymphocyte interplay that mediates the control of tumor growth.

## Introduction

After a long and intense debate there is now clear evidence that the immune system mediates tumor-suppressive effects in both humans and animals [1]. Using inbred mice with specific disruption of genes affecting critical components of the immune system or by selective ablation of immune cells and cytokines it has been shown that macrophages, natural killer T (NKT) cells, αβ T cells, γδ T cells, and natural killer (NK) cells as well as deletion or neutralization of cytokines such as interferon gamma (IFN-γ) and interleukin (IL)-12 all contribute to increased susceptibility of the host to develop tumors [2–6]. There is also evidence for cancer immunosurveillance in humans. Immunosuppressed transplant patients and individuals with immunodeficiencies have a significantly increased incidence of tumor development [7–9]. Additionally, immune checkpoint blockade has also been confirmed to be able to stimulate the immune system thereby mediating remarkable tumor remissions in subgroups of patients [10, 11]. These therapeutic responses are highly heterogeneous when comparing different tumor entities. Checkpoint blockade with programmed death-1 (PD-1) or programmed death ligand-1 (PD-L1) blocking antibodies induce tumor remissions and durable responses even upon treatment discontinuation in patients with melanoma, mismatch repair-deficient cancers, lung cancers and a variety of other malignancies [12–14]. The most reliable marker today to predict antitumor responses is upregulated PD-L1 expression within the cancer tissue [15, 16]. A high mutational load appears to facilitate immune recognition that can be amplified by checkpoint blockade [17, 18]. Consequently, tumors that are associated with viral transformation such as Hodgkin lymphoma [19] or tumors that frequently exhibit insertion-and-deletion-derived neoantigens such as renal carcinoma [20] are among the tumor entities that respond well to checkpoint blockade. Unfortunately, basic causes predicting individual antitumor responses still remain unknown. However, it is generally accepted that tumor immunogenicity is a main driver for effective checkpoint blockade [21, 22]. Tumor immunogenicity is a general term that describes the ability of the immune system to discern healthy and malignant cells and to trigger innate as well as adaptive immune responses counteracting tumor growth in untreated hosts or upon immunotherapeutic interventions.

Among those immune cells that contribute to tumor suppression, αβ T cells have attracted the attention of tumor immunologists and clinical scientists. This cell type is the central component of the adaptive immune system and its presence within tumor tissue in human colon carcinomas is correlated with a significant survival benefit [23]. Furthermore, these cells drive potent tumor regression in late stages of cancer in mouse models [21, 24–26] and upon immunotherapeutic applications in patients [12, 27, 28].

Although CD4 and CD8 T cell responses to cancer have been extensively described, it is still poorly understood how regulatory mechanisms and crosstalk between T lymphocyte subsets influence tumor immunity. Understanding these molecular mechanisms of T cell regulation in depth will be vital for the rational design and improvement of immunotherapies for the treatment of cancer. In the following sections we summarize briefly the main fundamentals of T cell immunity against cancer and discuss their potential and limitations to control cancer outgrowth.

## Establishment of the term ‘cancer immunosurveillance’

When Paul Ehrlich presented his studies on carcinomas in 1908, he was one of the first scientists to come up with the idea that the immune system is capable of suppressing carcinogenesis, thus lowering the frequency of developing tumors [29]. In the following decades neither his hypothesis about athreptic immunity nor his results on tumor vaccinations were thoroughly pursued, probably due to the still unevolved field of immunology. In 1957 the hypothesis about cancer immunosurveillance was revisited by several scientists. Sir Macfarlane Burnet proposed that cancers possess new antigenic properties that can provoke antitumor immunity [30]. Lewis Thomas speculated that in long-lived organisms allograft rejection is rather a side effect of the immune system, whereas its main function could be the protection of the host from altered cells and malignancy [31]. At the same time, Prehn and Main described immunity to methylcholanthrene-induced sarcomas and a study performed by Klein and colleagues demonstrated resistance to this kind of tumors in autochthonous hosts [32, 33]. In 1968, Ingegerd Hellström found evidence that cellular and humoral components of the immune system mediated antitumor effects in patients. Despite these observations, most patients suffered from tumor progression, a finding termed “Hellström paradox” [34]. It describes the dichotomy of tumor-directed immune responses in cancer patients with obvious tumor progress. In the following years, Burnet and Thomas both assumed that lymphocytes play a major role in cancer surveillance [35]. However, the details of antigen presentation and recognition by T lymphocytes remained undiscovered at that time and were outlined only some years later by Zinkernagel and Doherty in 1974, when they published their pioneering work on the function of the major histocompatibility complex (MHC) [36, 37]. This was an essential discovery to understand adaptive cellular immunity to pathogens and malignant cells. It took another 20 years until conclusive evidence from mouse models became available which demonstrated the existence of cancer immunosurveillance [3, 38]. Apart from IFN-γ, lymphocytes were clearly identified to prevent primary tumor development: in a key study by Shankaran et al., chemically-induced sarcomas derived from immunodeficient RAG2^−/−^ (recombination activating gene 2^−/−^) mice were rejected in immunocompetent mice, while sarcomas obtained from wildtype mice successfully engrafted indicating reduced tumor cell immunogenicity when tumors were induced in immunocompetent mice [39]. This study also led to a refinement of the hypothesis of cancer surveillance to cancer immunoediting. The authors showed that lymphocytes not only suppress tumor growth but at the same time also shape the tumor towards low immunogenicity, due to the immunoselection that is applied by this effective extrinsic tumor suppressor system to a growing and often genetically instable tumor.

Since then, the overall interest in tumor immunology has reemerged and an increasing number of mouse models has been established to investigate tumor-suppressive mechanisms [40]. The existence of cancer immunoediting is now generally accepted and tumor immune escape is regarded as an emerging hallmark of cancer [41].

## The role of lymphocytes in tumor-suppressive mechanisms and tumor outgrowth

Intrinsic tumor-suppressive mechanisms of healthy cells are active before the immune system becomes involved in tumor surveillance [42]. Aberrant activation of oncogenes in normal cells induces a p53-dependent state of stable cell-cycle arrest called cellular senescence. Senescent cells exhibit a secretory phenotype called SASP (senescence-associated secretory phenotype) [43] that is characterized by secretion of various cytokines mediating the attraction of immune cells. These immune cells ultimately lead to clearance of senescent cells in a process termed senescence surveillance [6]. In this study of Kang et al., antigen-specific CD4 T cells showing a Th1-phenotype were detected that were directed against oncogenic ras-expressing pre-malignant hepatocytes. Senescence surveillance is restricted to the pre-malignant state and requires macrophages and CD4 T cells. Interestingly, it has been shown that CD8 T cells are not required in this mechanism as long as the pre-malignant cell can be identified by SASP. If senescence is no longer maintained due to the accumulation of additional genetic alterations, such as loss of p53-function, the cell finally becomes malignant, loses its SASP and is no longer recognized by senescence surveillance. At this point, CD8 T lymphocytes start to play a crucial role in mediating tumor growth control [44]. This mechanism minimizes the risk of CD8 T cell-driven autoimmunity, since CD8 T cells are only induced in the last stage of tumor development. Additionally, the reactivation of cellular senescence in tumors by Th1-cytokines derived from CD4 lymphocytes has been identified recently as an additional mechanism for controlling tumor growth [45].

**Fig. 1:**
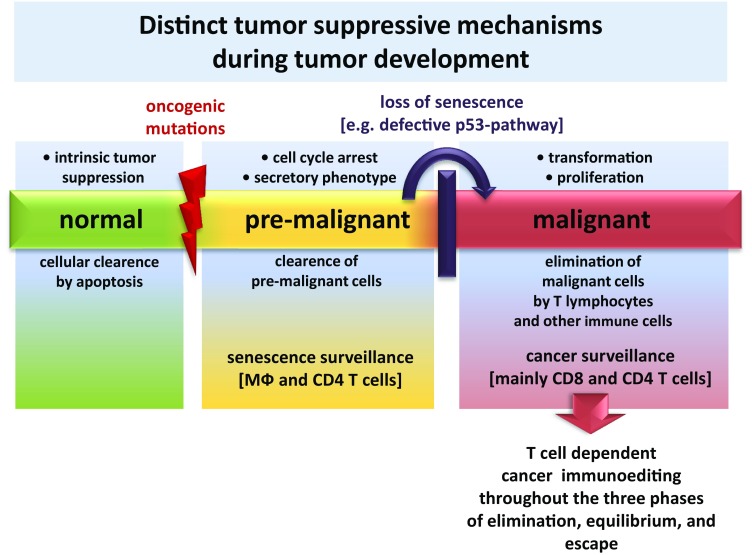
Diverse strategies of the host have evolved to facilitate tumor suppression in different stages of malignancy. A dispensable healthy cell or a cell that acquired severe damage usually succumbs to apoptosis, a mechanism that is required to maintain cellular homeostasis. This can be regarded as an initial barrier of tumor development. Aberrant cell cycle activation leads to cellular senescence and initiates the extrinsic mechanism of senescence surveillance that limits cancer development. CD4 T cells and macrophages are the essential immune cells that mediate senescence surveillance of pre-malignant cells. When cells escape senescence surveillance and further proceed in their course of tumor development, they become malignant and are then subjected to cancer surveillance. In this phase, CD4 and CD8 T cell responses play a central role in mediating the elimination of malignant cells. T-cell-mediated cancer surveillance furthermore leads to cancer immunoediting that shapes tumors towards low immunogenicity

Tumor outgrowth is mainly controlled by CD4 and CD8 T cells [39]. During tumor development cancer immunoediting occurs with its three phases namely elimination, equilibrium and escape which have been described in great detail in other reviews and are therefore only briefly described here [1, 9, 40, 46]. After cellular transformation, nascent tumor lesions trigger an immune response that specifically eliminates these lesions, thus protecting the host from cancer; this refers to the elimination phase. When, however, the immune response is incapable of completely clearing the tumor cells during the equilibrium phase, but still prevents tumor outgrowth, the process of incomplete elimination promotes the generation of tumor cell variants with decreased immunogenicity. The existence of this phase and the ability of the immune system to maintain occult cancer in an equilibrium state have been demonstrated by Koebel et al. in mice [47]. Furthermore, issues with transplantation and neoantigen landscape studies indicate that this state also exists in humans [9, 48]. Following this selection process, sculpted tumor cells with low immunogenicity expand to a clinically manifest tumor. Yet, there is evidence that the immune system does not shape tumor immunogenicity towards complete tolerance. When tumor-infiltrating T cells were isolated from tumor tissue, expanded in vitro, and then retransferred in lymphopenic patients, this method showed impressive clinical effects [49]. The term “cancer immunosurveillance” is more applicable to early stages of host protection from neoplastic diseases. The concept of cancer immunoediting comprises not only events of early stages in tumor development, but also the consequences of this process on shaping the immunogenicity of a growing tumor. Figure 1 illustrates strategies of the host to facilitate tumor suppression throughout the whole course of tumor development. Furthermore, the graphic shows immune cells of vital importance for tumor-suppressive functions as described above in the pre-malignant and malignant state during tumor development.

By the time a malignant disease becomes apparent, the tumor has escaped intrinsic growth control, senescence surveillance, and additionally, the tumor has reached the final state of cancer immunoediting, thus it has been sculptured towards low immunogenicity. The process of immunoediting has considerable impact on shaping the neoantigenic landscape of tumors for induction and expansion of T cell immune responses that determine accessibility to immunotherapies [17, 48, 50, 51].

Apart from T cell-induced selection of tumor cells towards those with reduced immunogenicity, another important mechanism affecting tumor-suppressive T cell responses is the induction of a dysfunctional state, anergy or even apoptosis of these cells, that can also have detrimental effects in immunotherapies [52]. Under certain conditions, suppression of cytotoxic T lymphocytes (CTLs) can be established upon direct cell-to-cell contact with their corresponding target cell. Tumors are capable of suppressing T cells once they migrate into tumor margin or infiltrate tumor tissue where they encounter the tumor microenvironment (TME). The TME consists of tumor cells, non-malignant stromal cells including the tumor vasculature, and stroma-infiltrating immune cells. Highly dynamic cell signaling networks, cross-talks, and adaptations within these components of the TME during tumor progression often confer resistance to tumor immunity and negatively influence therapeutic regimens by numerous mechanisms [53, 54]. A recent study by Katlinski and colleagues investigated the resistance of colorectal cancer (CRC) to CTL-mediated growth control [55]. They found that CRC cells downregulate the type I interferon receptor chain IFNAR1 in the stroma thus generating an immune-privileged niche. IFNAR1 regulates the viability of CTLs, and a low expression of this receptor confers TME stress on lymphocytes, leading to apoptosis. Other immunosuppressive mechanisms of the TME affect tumor-directed T cell responses and drive them to T cell exhaustion, a mechanism that has evolved avoiding immunopathology and is exploited by tumors to escape the immune attack. Exhausted T cells lose effector function and upregulate inhibitory receptors (reviewed in Refs. [56, 57]). This state is frequently associated with a loss of tumor growth control. Interestingly, exhaustion can be reversible. Using antagonistic antibodies targeting the exhaustion markers T cell immunoglobulin and mucin domain containing protein 3 (Tim-3) and PD-1, it has been shown that T cells can regain effector functions [58]. This has a particular clinical impact, because PD-1 blockade alone can lead to emergence of resistant tumor cell clones against immunotherapeutic treatment by loss-of-function mutations in genes encoding the interferon-receptor-associated Janus kinase 1, Janus kinase 2, or the gene encoding the antigen-presenting protein beta-2-microglobulin [59]. Some mutations also endow cancer cells with features that make them innately resistant against PD-1 blockade [60]. Development of PD-1 resistant tumors seems to involve other immune checkpoints such as Tim-3 that additionally regulate tumor directed T cell responses [61].

In models of chronic viral infections with antigen persistence much progress has been made understanding the regulation of exhausted CD8 T cells (Tex). In a recent study, Pauken et al. analyzed the cellular, transcriptional, and also epigenetic changes upon checkpoint blockade in a mouse model of chronic lymphocytic choriomeningitis virus (LCMV) infection [62]. Here, PD-L1 blockade led to a reinvigoration of Tex and induced a transcriptional re-engagement in the regulation of effector functions. However, this reinvigoration was lost again when antigen concentration remained high. Furthermore, these re-exhausted cells failed to generate memory T cells (Tmem) after antigen clearance. The results suggest that Tex is a distinct lineage of CD8 T cells. Most importantly, the authors indicate that epigenetic fate inflexibility may have limitations for immunotherapies, but moreover, treatment with IL-7 during the effector phase is able to prevent the development of Tex. Further investigations will reveal whether these findings will have impact on current immunotherapies of cancer. Sen et al. performed an in-depth analysis of the epigenetic landscape of T cell exhaustion [63]. To this end, the authors used chronic LCMV infection in mice to generate Tex and compared chromatin-accessible regions from Tex with functional Teff. This approach identified differential regulatory regions between the two groups that showed features of enhancing elements. Tex acquired a state-specific epigenetic landscape organized into functional modules of enhancers. In this study, this finding could also be validated in human antigen-specific CD8 T cells from subjects with chronic progressive HIV-1. Knowledge of functional enhancer maps may lead towards the development of regimens exploiting genome editing to alter gene expression in exhausted CD8 T cells. Thus, combining current checkpoint blockades with genome editing strategies to maintain T cell immunity to cancer cells would be an elegant approach to avoid acquired therapy resistance due to T cell exhaustion.

While T cell exhaustion for CD8 cells is quite well described, much less is known about exhaustion of CD4 T cells in cancer. Nevertheless, the phenotype of T cell exhaustion seems to occur in both T cell populations, as described in a rodent cancer model [64]. In another preclinical study by Goding et al., tumor-specific CD4 T cells were investigated and showed considerable upregulation of several co-inhibitory receptors (PD-1, TIM-3, lymphocyte-activation gene-3 (LAG-3), 2B4 and T cell immunoreceptor with Ig and ITIM domains (TIGIT)), accompanied by reduced effector cytokine expression [IFN-γ and tumor necrosis factor-α (TNF-α)] [65]. It should be noted that this dysfunction-associated phenotype occurred only in mice with relapsing melanoma and suggests that exhaustion of tumor-specific CD4 T cells contributed to recurrence of melanoma. In a clinically relevant approach, the researchers highlighted the necessity of combination therapy for the treatment of relapsing melanoma. The treatment of recurrent melanoma by combined checkpoint blockade against PD-L1 and LAG-3 turned out to be highly effective. This treatment led to a reversal of CD4 T cell exhaustion and restored the effector functions of tumor-specific CD4 T cells.

Cancer-induced T cell exhaustion consequently contributes to tumor progression by quenching remaining responses derived from residual tumor immunogenicity of immunoedited tumors. In addition, the aforementioned low immunogenicity of cancer cells is not always a result of immunoselection, but may also derive from a lack of suitable target structures for T cell recognition. As tumor-directed CD4 and CD8 T lymphocytes belong to the adaptive arm of the immune system they can only develop in dependence on their corresponding antigens. This makes tumor-specific antigens (TSAs) to be decisive molecular determinants for tumor growth control and the fundamental source of all T cell responses against cancer.

## Immune targeting of cancer: tumor-specific antigens of CD4 and CD8 T cells

The major histocompatibility complex (MHC) is a highly variable region of the mammalian genome that encodes a large number of different loci comprising genes with many allelic variants [66]. The MHC class I and II genes of this polymorphic region encode the major proteins that are required for antigen presentation on the cellular surface. All MHC molecules share a common structure of a binding groove for peptides of different origin. Whereas all nucleated cells express MHC class I that bind peptides from degraded proteins of endogenous origin, MHC class II presents peptides of exogenous origin and is predominantly expressed by antigen-presenting cells, such as macrophages, B cells, dendritic cells, Langerhans, and Kupffer cells [67]. Some cells can show MHC class II expression under anomalous conditions, such as thyrocytes and intestinal epithelial cells in inflamed tissue [68, 69], as well as cancer cells [70, 71]. Furthermore, MHC class II expression can also vary depending on the location of the tumor cell: expression levels can be high in primary tumor lesions, but are often absent in metastases [72]. Abundant expression of MHC class II can also lead to potent tumor-specific CD4 T cell responses that dampen cytotoxic CD8 T cell responses in a TNF-related manner [73]. However, most tumor cells do not express MHC class II at all [74]. This has important implications for associated mechanisms of tumor-directed CD4 T cell responses that will be discussed in the next section. Preclinical studies investigating the role of CD4 T cell responses in MHC class II-positive tumors were performed in mouse models using the B16 melanoma cell line raising the question whether CD4 T cell responses are similarly regulated in other melanoma models and tumor types. Homet et al. addressed this issue by generating BRAF^V600E^-driven YUMM1.1 and YUMM2.1 cell lines derived from genetically engineered mice with melanocyte-specific mutant BRAF and PTEN knockout [75]. The authors comprehensively characterized the immunological properties of this model upon PD-1 blockade. Compared to the PD-1-responsive colon cancer control cell line MC38, both YUMM cell lines showed a considerably lower mutational load and distinct responsiveness to PD-1 immunotherapy. Antitumor activity was observed in YUMM2.1, but not YUMM1.1. In contrast to MC38, antibody-mediated depletion of CD8 cells only partially rescued therapeutic efficacy of PD-1 in YUMM2.1. Depleting YUMM2.1-bearing animals of CD4 cells completely abrogated the response to PD-1, suggesting a prominent role of CD4 T cells in this melanoma model. Although the MHC class II status of YUMM2.1 remains unknown, the results show differential immunological features to checkpoint blockade compared to B16 melanoma models. Also in other malignancies, apart from melanoma, MHC class II expression on tumor cells crucially affects tumor immunity. In a murine model of pancreatic ductal adenocarcinoma (PDA), two different cell lines were transduced with MHC class II transactivator (CIITA) and injected into syngeneic mice [76]. CIITA-positive PDA tumors were rejected, long-lasting memory responses were established, and recruitment of T cells to the tumor area was evident. In humans it has been shown that the magnitude of CD4 T cells in microsatellite-unstable (MSI-H) colon carcinomas is significantly higher in HLA class II-negative tumors harboring mutations in HLA class II-regulatory genes such as RFX5, CIITA, and RFXAP [77]. These data suggest that lacking HLA class II-expression on MSI-H colon carcinoma cells favored tumor progression in an environment of dense CD4 T cell infiltration. Taken together, these studies indicate that expression of MHC class II on tumor cells and corresponding CD4 T cell responses behave differently not only in a variety of tumor entities, but also in tumors of similar origin.

The frequency of tumor-specific MHC class II epitopes is usually much higher compared to MHC class I epitopes [78, 79]. This may be explained by the stringent sequence requirements and the defined length of peptides binding the MHC. MHC class II molecules have an open binding groove and peptides can protrude on both sides, making the length of binding peptides more flexible than MHC class I molecules, where the closed binding groove mainly binds peptides of 8–10 amino acids. Moreover, MHC class I is essential for CTL-mediated tumor elimination. It is therefore frequently downregulated in tumors, but a complete loss is a rare event, probably due to other counter regulations of the immune system [80] such as recognition of MHC I-depleted cells by NK cells.

Tumor-derived peptides that significantly shape tumor immunity can be divided into five classes of TSAs [81–83]: *Overexpressed antigens* (e.g. Human epidermal growth factor receptor 2 (HER-2/neu)); *differentiation antigens* (e.g. melanocyte differentiation antigens); *mutational antigens*, the vast majority of mutations are unique to each patient, hence the neoantigenome has a largely individual pattern; v*iral antigens* [e.g. derived from Epstein–Barr virus (EBV)], and *cancer*-*testis antigens* [e.g. New York esophageal squamous cell carcinoma-1 (NY-ESO-1)]. Expression of cancer-testis antigens in adults is limited to germ cells in the testis. Tumor-associated re-expression of this class of antigens can be found in many tumor entities. Cancer-testis antigens are of particular interest for immunotherapy [84]. The classes of non-mutated antigens are associated with immune infiltrations and increased survival in patients with hepatocellular carcinoma [85, 86]. However, complete clinical remissions based on self-antigens have not been observed so far.

The TSAs that recently have come into focus are mutational antigens. Early studies using molecular methods already suggested that non-synonymous mutations are ideal candidates for the immune system to identify altered cells. Wolfel et al. identified a p16^INK4a^-insensitive cyclin-dependent kinase (CDK4)-R24C mutant in tumor cells of a melanoma patient. The epitope of this mutant form was targeted by CTLs and it was confirmed to occur in another cohort of melanoma patients. This mutation of CDK4 can generate a TSA and disrupt the cell-cycle regulation exerted by the tumor suppressor p16^INK4a^ [87]. Later, it has been shown that the response of autologous T cells in melanoma is predominantly driven by mutated neoantigens [88]. Recognition of these neoantigens by the immune system is important for the therapeutic efficacy of checkpoint inhibitors [89]. Consistently, it has been shown that T cell responses raised by immune checkpoint blockade are mainly directed against mutant antigens [21, 27]. It has also been demonstrated that PD-1 is mainly upregulated on melanoma-specific T cell clones with high functional avidity [90]. In contrast, low avidity T cell clones had a substantially methylated PD-1 promotor region indicating epigenetic regulation of PD-1 expression and maintenance of peripheral blood cells in presence of PD-1 blocking antibodies led to selective expansion of high avidity T cell clones.

The effectiveness of immunotherapies against neoantigens has been convincingly demonstrated by induction of therapeutic CD4 and CD8 T cell responses in mouse models of melanoma, sarcoma, and colon cancer [21, 26, 91]. Therefore it is very likely that neoantigens will maintain their key role in future strategies of personalized immunotherapeutic applications.

In silico approaches of cancer neoantigen prediction have been used based on genomic sequencing data to generate putative peptide:MHC binders, such as SYFPEITHI [92] and NetMHC [93]. Using such prediction algorithms, identification of high affinity neoepitopes has been very successful in several studies [21, 25, 27, 50, 91]. It is known from reductionistic models using adoptive T cell therapies that binding affinities between antigenic peptides and MHC class I and also the binding affinity of the peptide:MHC complex to the corresponding T cell receptor (TCR) are critical determinants of antitumor T cell reactivity and the capability of T cell responses to reject a tumor [94]. However, there are several caveats to focus on single high affinity binding epitopes. First of all, neoepitope patterns are more or less unique among cancer patients with very little overlap. Immunotherapies such as DC-vaccinations targeting a specific neoepitope are therefore at best limited to a small number of patients. Even if more common neoepitopes bind to MHC class I with high affinity it is likely that such epitopes have undergone high selection pressure during immunoediting. Consistently, potent T cell responses against a particular epitope can also promote the occurrence of antigen-loss variants by epigenetic gene silencing of protein expression [95]. A study of Zhong et al. suggests that binding affinities above a certain threshold do not necessarily improve efficacy [96]. Additionally, neoepitope-directed CD8 T cell responses of lower affinity have also been shown to play a role in immunotherapeutic applications when regarding polyvalent responses [97, 98]. These responses would probably be best suited to prevent the generation of escape variants by the tumor. Future investigations will have to address the relevance of binding affinities in cancer neoepitope prediction in relevant models.

Taken together, occurrence of tumor-specific CD4 and CD8 T cells in tumor tissue is regarded as a good prognostic factor [23, 99], but tumor immunogenicity is not a general characteristic of tumor development [100]. Induction of tumor immunogenicity is a major aim in immune checkpoint blockade that is still achieved infrequently [15, 101].

## T helper cell polarization, direct and indirect mechanisms of tumoricidal CD4 T cells, and regulatory T cells

So far we have discussed the frame conditions in which T cell responses against cancer cells arise. CD8 T cell responses are usually regarded as representing the immune cell type of paramount importance for control of tumor growth due to their potent cytotoxicity and the observation that tumors can prevent elimination by downregulation of MHC class I expression [102, 103]. In contrast, the role of CD4 T cell immunity to cancer is much less understood. It has now become increasingly clear that tumor-specific CD4 T cells display a complex biology and their roles are far beyond the mere task of providing helper signals to CD8 T cells [104]. Naïve CD4 T cells are able to differentiate into multiple effector subsets that can mediate various, even opposing functions. In one of the first studies showing that CD4 T cells have significant antitumor effects, Greenberg and colleagues performed an adoptive transfer of T cells obtained from syngeneic mice vaccinated with irradiated tumor cells that facilitated eradication of disseminated leukemia in recipients [105]. Within the last three decades several studies followed and further unraveled the mechanisms of CD4 T cells in tumor immunity.

The predominant helper cell subtypes are Th1 and Th2. Th1 commitment depends on local IL-12 secretion, whereas Th2 cells arise in dependence on IL-4 and in the absence of IL-12. Th2 cells have been reported to exert antitumor effects [106, 107]. Recently, it has been demonstrated that adoptively transferred Th2 cells were able to eradicate subcutaneous MHC class II-negative myeloma in mice [108]. The authors showed that persistence of Th2 cells in vivo correlates with long lasting immunity. The observed eradication of the tumor occurred in an antigen-specific manner and was independent of B cells, NK cells, CD8 cells, and IFN-γ. Transferred Th2 cells were able to induce a type II inflammation at the tumor site that interestingly involved M2 macrophages, which are usually regarded as a tumor-promoting macrophage subtype. Together these results highlight the potential of adoptive transfer of antigen-specific Th2 cells. Nevertheless, Th1 cells are regarded as the most important helper cell type for cancer immunity, being involved in the killing of tumor cells by secretion of cytokines that activate death receptors on the tumor cell surface and in the induction epitope spreading [109]. Th1 cells are also able to activate DC cytotoxic functions that eliminate tumor cells in an IFN-γ-dependent manner and provide a source of tumor-associated antigens derived from the killed tumor cells [110]. Furthermore, beside IFN-γ, cancer-specific CD4 T cells can also secrete IL-4 [106, 111–113], establish long-term memory immune responses to tumors [114, 115], and recruit eosinophils and macrophages [116, 117]. The study performed by Corthay and colleagues also comprehensively characterized the mechanism by which MHC class II-negative myeloma cells were identified and eliminated by CD4 T cells [117]. In this study, T cells from TCR-transgenic donors with a SCID-background were transferred to recipient mice and tumor challenge led to an activation of tumor-specific CD4 T cells in the draining lymph nodes. These T cells migrated to the tumor where they massively recruited and activated macrophages. CD4 T cell-depletion completely blocked macrophage activation. Moreover, MHC class II-blocking antibodies impaired the activation of tumor-specific CD4 T cells in the draining lymph nodes and inhibited migration to the tumor site and the activation of macrophages. In further experiments the authors injected TSA-positive myeloma cells in the right flank of mice and a TSA-negative control cell line in the left flank. Tumor-specific CD4 T cells became activated in the draining lymph nodes of myeloma tumors, but not in lymph nodes of the control tumors. This showed that the primary activation of tumor-specific CD4 T cells is locally restricted to the tumor site, rather than a systemic phenomenon. Lastly, the study again stressed the importance of IFN-γ in this mechanism of immunosurveillance that is released upon collaboration of tumor-specific CD4 T cells with macrophages. The indirect recognition mechanism of Th1-polarized CD4 T cells has been comprehensively investigated in a melanoma model as well [118]. Here, a direct killing was abolished by using an MHC class II-disparate model and clearance of tumor cells by CD4 T cells was critically reliant on IFN-γ. In general, these studies demonstrate that tumor-specific Th1-polarized T cells are capable of indirect tumor cell elimination by involving assistance of macrophages.

Another helper cell commitment distinct from Th1 and Th2 is the Th17 lineage, which is induced by TGF-β and IL-6 [119, 120]. T helper cell type 17 in mice and men have been associated with the production of IL-17 [121, 122] and expression of transcription factor retinoic acid-related orphan receptor gamma t (RORγt) [123, 124]. It has also been described that cyclophosphamide induces differentiation to Th17 cells in rodents and cancer patients [125]. This lineage has been associated with infectious diseases and development of experimental autoimmune encephalitis and collagen-induced arthritis in mouse models [126, 127]. Naturally occurring Th17 cells have also been identified in association with certain kinds of cancer, such as prostate cancer and cutaneous T cell lymphomas [128, 129]. A work conducted by Muranski et al. demonstrated eradication of an established melanoma by tumor-specific Th17 that was critically dependent on IFN-γ [71]. In this study Th0, Th1, and Th17 cells were generated in vitro and then adoptively transferred to tumor bearing mice. In contrast to Th17 cells, Th1-polarized cells were capable of secreting high levels of IFN-γ, but the investigators found that Th17 cells were superior in mediating destruction of advanced melanomas. It can only be speculated, why Th17 cells are highly dependent on IFN-γ. Indeed, Th17 cells secrete, apart from IL-17 and TNF-α, IFN-γ upon in vitro stimulation in this study and in models of autoimmunity [121], but the underlying mechanism of cytokine interplay for tumor rejection and autoimmunity is to be elucidated. Another important aspect of Th17-polarized T cells is their ability to mediate direct cytotoxicity on MHC class II-positive tumors [111, 112]. Adoptively transferred T cells from TCR-transgenic mice using Trp1-specific T cells in the context of MHC class II I-A^b^ have been used to demonstrate that these cells are able to eradicate B16 melanoma cells through a Granzyme-dependent mechanism [130]. As already mentioned, the recognition of non-secreted MHC class II antigens by CD4 T cells is mediated by macrophages. However, it has been shown that this is not sufficient to elicit direct cytotoxic effects of CD4 T cells [131].

In Table 1 we compare the key observations of animal experiments with the results of clinical studies investigating antitumor effects of Th2 and Th17 cells. While some of the key results are compatible with each other regardless of the host, there are also some other findings with remaining discrepancies. For example, while Th2 cells have been repeatedly shown to eradicate tumors in murine models [106–108], they have been observed to contribute to chronic inflammation in patients with metastases in clinical studies [132]. If Th2 responses are elicited in consequence of alternatively activated macrophages in the tumor tissue, it promotes the formation of an immunosuppressive microenvironment as shown in patients with esophageal cancer [133]. This was a central issue in a recent study by Ito et al., showing that blocking IL-4 changes the microenvironment of the tumor to the favor of Th1-polarization from Th2 in mice, suggesting a possible enhancement of human immunotherapy by IL-4 mAb treatment [134]. Pharmacologic interventions can also have a significant effect on Th17 responses in mouse models [135]. The use of a Th17 inhibitor SR1001 decreased the formation of micro-invasive prostate cancer in Pten-null mice. Furthermore, the compound also showed additional antitumor effects suggesting that Th17 responses can have a tumor supportive role. Also in humans there have been seemingly conflicting reports with regard to IL-17 expression by cells in tumor tissue and survival of patients [136, 137]. It is possible that the contribution by Th17 cells during the antitumor response and promotion of tumor growth is dependent on the cancer type or even the immune status of the host [138, 139]. In ovarian cancer [140], esophageal squamous cell carcinoma (ESCC) [141], head and neck squamous cell carcinoma (HNSCC) [137] the clinical data suggest Th17 cells to be involved in tumor suppression. In the latter study the data implicates Th17 cell migration towards the tumor site where these CD4 T cells inhibit the angiogenesis and suppress the proliferation of cancer cells. Additionally, a correlation between increased tumor-infiltration by IFN-γ producing CD4 and CD8 T cells, and the increased frequency of Th17 cells in the tumor tissue could be detected [140]. In contrast, higher numbers of IL-17 producing cells in breast cancer tissue are associated with shorter disease-free survival of patients [142]. Also the intratumor- and peritumor-enrichment of IL-17 producing cells is associated with shorter survival of patients with HCC [136].

**Table 1:** Comparing observations of animal studies with clinical evidence involving the antitumoral effects of Th2 and Th17 cells

Tumor type and experimental model	Treatment/model	Observation	References
Th 2 cells			
B16 and B16-OVA melanoma model	Tumor established in C57BL/6 mice	Th2 cells eradicate melanoma metastases in a STAT6 and eotaxin-dependent manner	[107]
Human, metastatic melanoma	N/A	Th2 drives chronic inflammation in these patients	[132]
Murine B cell lymphoma cell line	Tumor established in BALB/c^106^ or SCID^108^ mice	Th2 cells producing high levels of IL-4 eradicate the tumor	[106, 108]
Murine mammary carcinoma and colon cancer cell line	Tumor established in CNS2 KO mice, anti-IL-4 Ab administration	IL-4 blockade causes a shift in the tumor microenvironment from a Th2- to Th1-polarization	[134]
Human, esophageal cancer	N/A	Increased expression of IL-4	[133]
Th 17 cells			
B16-F10 melanoma model	Cyclophosphamide administration	IL-17 and IFNγ increase and induction of a Th17 pool	[125]
Advanced cancer patients	Cyclophosphamide administration	IL-17 increase and induction of a Th17 pool (no significant effect on IFNγ)	[125]
Pten-null mice	Th17 inhibitor SR1001 or anti-mouse IL-17 mAb	Therapeutic decrease in the formation of micro-invasive prostate cancer	[135]
Human benign hyperplastic, and prostate carcinoma	N/A	IL-17 increase	[128]
Human cervical tumor cell line	Tumor established in nude mice	Tumor size increase of IL-17 expressing cervical cells in immunodeficient mice	[138]
Murine plasmocytoma and mastocytoma model	Tumor established in immunocompetent mice	IL-17 transfection inhibits hematopoietic tumor growth in immunocompetent mice	[139]
In vitro polarized tumor-specific T cells	Adaptive transfer into tumor bearing mice of the B16 melanoma model	Th17 cells eradicate the tumor in a INFγ-dependent manner	[71]
Human study, cutaneous T cell lymphomas	N/A	IL-17 increase	[129]

In fact, the characterization of Th17 cells based on IL-17 expression alone can be delusive, since IL-17-expressing T cells in human blood and lymphatic tissue can also belong to immunosuppressive regulatory T cells (Tregs), a T cell lineage that will be introduced shortly [143]. Voo et al. could demonstrate that IL-17 expression was not limited to Th17 cells and suppressive Tregs expressed both lineage transcription factors RORγt (Th17) and forkhead box P3 (Foxp3). They further showed that the suppression of CD4 T cell proliferation could be in vitro induced by IL-17 positive Tregs and IL-17 negative Tregs, likewise. Furthermore, evidence suggests that a conversion of Th17 cells into immunosuppressive regulatory T cells is possible in the tumor microenvironment [144]. This finding may reflect the increased frequency of IL-17-producing cells in HCC, a cancer type where accumulation of Tregs is associated with cancer recurrence [145]. Overall, the plasticity of Th17 cells and seemingly contradictory findings in various cancers necessitate more detailed characterizations of this T helper cell type.

A CD4 T cell subtype that has been identified to play an important role in maintaining immunological tolerance to self- and non-self-antigens are regulatory T cells. Depletion of these cells causes several autoimmune disease-phenotypes in mice. Tregs are characterized by expression of the IL-2 receptor alpha chain (CD25) that was identified as a central marker by a seminal study of Sakaguchi et al. [146]. In addition, the forkhead transcription factor Foxp3 was later identified as an important regulatory transcription factor of Tregs [147, 148]. Upon characterization of these molecular markers, the link between Tregs contributing to tumor development and suppression of antitumor immunity was clearly established [149–151]. During cancer progression, an increase of Tregs was frequently observed independently of the thymus [152] in blood and tumor margins that suppress CD4 and CD8 T cell responses as well as dendritic cell function [153, 154]. Tregs located in the tumor microenvironment mediate immunosuppression by releasing cytokines such as TGF-β and IL-10 [155].

Several studies describe a conversion of conventional CD4 T cells to regulatory T cells in the tumor microenvironment. There is discordance whether thymus-derived Tregs mediate the conversion of conventional CD4 T cells to immunosuppressive Tregs by TGF-β and IL-10 [156, 157] or if peripherally-derived Tregs are accumulating in the tumor tissue [158, 159]. Regardless of their origin, thymus-derived Tregs and peripherally-derived Tregs independently contribute to tolerance in the tumor environment [160]. The infiltration of tumors by CD8 T cells is mostly considered as beneficial for the survival of the patient and the ratio of CD8 T cells/Tregs is regarded as a crucial prognostic factor for different types of cancer. A higher ratio of CD8 T cells/Tregs is usually associated with a favorable outcome as demonstrated in ovarian and liver cancer [145, 161]. A shift in the balance of tumor-infiltrating lymphocytes towards higher numbers of Tregs is associated with a poor prognosis in gastric and breast cancer [162, 163]. On the other hand, lymphomas and colorectal cancer account for malignancies in which occurrence of high numbers of Foxp3-positive Treg cells is associated with a good prognosis [164–167]. Unfortunately, the underlying mechanism of immune regulation of this opposing role for Tregs is not yet known.

A number of studies have already assessed suppressive mechanisms of Tregs. There is convincing evidence that the co-inhibitory receptor cytotoxic T lymphocyte antigen 4 (CTLA-4) is a potent mediator of Treg-induced immunosuppression [168]. In particular, during the interaction of Tregs and antigen-presenting cells, CTLA-4 is able to bind with high avidity to the CD80 and CD86 molecules that are present on the surface of antigen-presenting cells (APCs) [169]. This interaction leads to down-regulation and capture of CD80 and CD86 by trans-endocytosis from APCs, thereby depriving CD8 T cells of co-stimulatory signals that are required for sufficient activation of T cells [170, 171]. This process is mediated by CD80/CD86 ligation to the co-stimulatory CD28 receptor expressed on CD8 T cells. The impact of preventing interference by CTLA-4 has been demonstrated in melanoma patients where CTLA-4-blockade induced a broadening of tumor directed T cell responses [172]. Although the relevance of Tregs is mostly considered during initial T cell priming whereby Tregs inhibit T cell activation by CTLA-4 intervention, there is also evidence that Tregs apply other mechanisms to enforce peripheral tolerance in the tumor environment. In a model of acute myeloid leukemia (AML) only wild-type Tregs prevented tumor rejection, compared to Granzyme B knockout (*Gzmb* ^−/−^) or Perforin1 knockout (*Prf1* ^−/−^) Tregs [173]. The regulatory T cells from the tumor microenvironment were also able to induce cell death of NK cells and CD8 T cells that required Granzyme B and perforin. In a different cancer model Bauer et al. highlighted that antigen recognition by Tregs in the tumor tissue is necessary to prevent tumor rejection [174]. These mechanisms of immunological tolerance induction reflect the role of Tregs in the tumor microenvironment by shifting the CD8 T cell/Treg ratio in favor of immunosuppression in various cancer types.

Furthermore, therapeutic agents have been described to influence the Treg-population: in cancer patients IL-2 increases the amount of Tregs [175, 176] and prostaglandin E2 can be used to induce Foxp3-expression and to increase suppressive activity of CD4 + CD25 + cells [177]. In contrast, low-dose cyclophosphamide eradicates Tregs and can thereby sensitize tumors to immunotherapy [178]. Hence, therapeutic interference with Treg-activity appears to be an important strategy to overcome immunosuppression of T cell responses in various immunotherapeutic applications.

Taken together, these studies show the complex regulation of immune responses by CD4 T cells that mediate antitumor immunity or promote tumor growth, depending on the context and the applied regimens. CD4 T cells are able to communicate with several types of immune cells and other cells of non-hematopoietic origin. Thus, they are a key component for modulation of tumor immunity. Finally, functional CD8 T cells are strictly dependent on CD4 T cell responses against cancer, a point that is often neglected in studies investigating cytotoxic CD8 T cell responses.

## The interplay of CD4–CD8 T cells in controlling tumor growth

**Fig. 2:**
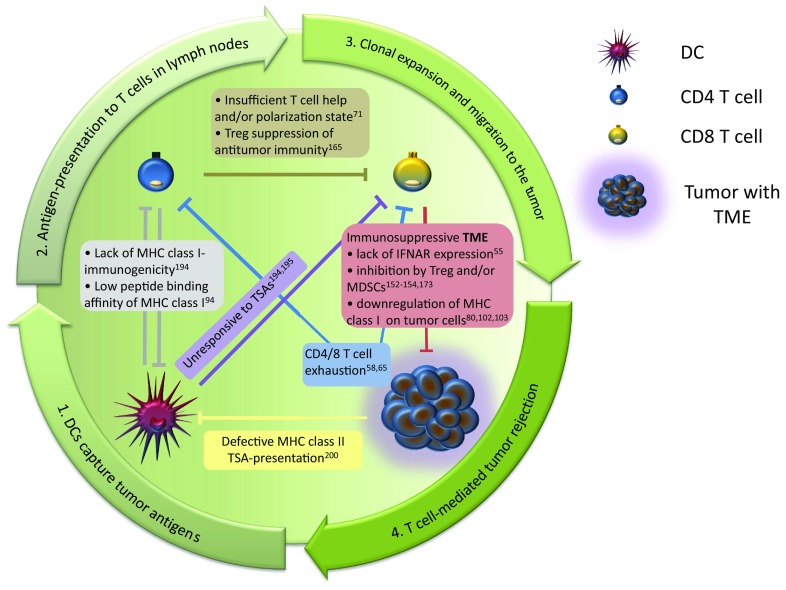
Failure of tumor rejection is often due to a malfunction within any step of the cancer immune cycle. Central components of this cycle are CD4 and CD8 T cells that are involved in all steps of the cycle. Crucial steps of the cancer immune cycle are outlined by the arrows. Any disruption of mutual CD4/CD8 T cell interplay or other crucial steps of T-cell-signaling within this circle that have been demonstrated to abort the whole cancer immune cycle are noted within the graphic representation. The figure comprises studies that have been discussed in this review and therefore does not provide a complete overview. All descriptions include the reference to original studies

For cellular immunity the mutual relationship between CD4 and CD8 T cells for tumor suppression is a special feature of the immune system, since interactions between CD4 and CD8 T cells both derive from the adaptive arm of the immune system and require antigen experience for both T cell populations to trigger antitumor immunity. In a broader sense, the crosstalk of these T lymphocytes is part of the cancer immune cycle [179, 180]. This cycle describes the sequence of events that leads to the generation of tumor-directed T cell responses induced by dendritic cells that have captured TSAs, migrate to the lymph nodes and present their antigens to T cells. Tumor-specific T cells clonally expand and subsequently migrate through the lymphatic vessels and other tissues to the tumor, where malignant cells are successfully eliminated by these T lymphocytes. In the context of immunotherapies, all combined factors influencing the cycle of this complex process have also been described as the ‘cancer-immune set point’ [181]. Only a fully completed cycle enables the immune system to counteract tumor growth. To that effect, any interruption or defect within a single step of this cancer immune cycle can lead to failure of the whole process allowing the tumor to escape immune control. In this regard, the same rule is applicable to the CD4 and CD8 T cell interplay, as being part of the cancer immune cycle: any disruption of crucial cross-talk events results in a failure of tumor growth control. Incidents that can lead to a failure of the cancer immune cycle due to erroneous CD4 and CD8 T cell signaling are shown in Fig. 2.

Several concepts and mechanisms for T cell crosstalk derive from mouse models using pathogens. Whereas rapid T cell contraction is generally observed after pathogen infections, frequently observed ongoing responses upon treatment discontinuation of immunotherapies in cancer patients challenge the view of the consistency in the regulation of pathogen- and cancer-derived T cell responses [11, 182]. Whether conclusions based on such pathogen models are generally applicable for cancer immunology is therefore an issue that needs further attention. The obvious differences in the requirements for the immune system to mediate pathogen clearance and to drive tumor remissions also suggest differences in immune regulations and T cell phenotypes. Accordingly, there is a general ambition to summarize the characteristics of cancer-specific T cells [183].

Also cells of non-hematopoietic origin appear to play a crucial role in cytokine-mediated tumor regression. Numerous studies emphasize the importance of IFN-γ in tumor suppression, a cytokine that is frequently released by activated CD4 and CD8 T cells. A recent study unraveled the effect of IFN-γ on the TME and identified cells of the tumor stroma that are involved in the rejection of large, established tumors [184]. Whereas T cells, other hematopoietic cells, or fibroblasts were not sufficient to induce IFN-γ-induced tumor regression, Kammertoens et al. showed that responsiveness to IFN-γ of endothelial cells was necessary and sufficient in this model. Further analysis revealed IFN-γ-induced regression of the tumor vasculature that was similar to non-hemorrhagic necrosis in ischemia. These results underline the complex regulation of the IFN-γ-pathway and suggest that T cells, as a source of IFN-γ, can also contribute to tumor remissions mediated by endothelial cells.

Until now only few studies investigated the contribution of both CD4 and CD8 T cell responses in tumor models with autologous immunity. Pioneering in vitro studies that addressed CD4/CD8 T cells interplay emphasized the importance of both populations for tumor immunity [185, 186]. The use of TCR transgenic mice with specificities for MHC class I and II as a source of naïve HA-specific T lymphocytes in a model of malignant mesothelioma showed a greatly enhanced T cell response and tumor rejection when CD4 and suboptimal numbers of CD8 lymphocytes were co-transferred, whereas adoptive transfer of CD8 T cells alone was not sufficient to induce tumor remissions [187]. Consistently, Church and colleagues demonstrated that tumor-specific CD4 T cells help to maintain functionality of tumor-directed CD8 T cells [188]. They observed that CD8 T cells expressed lower levels of PD-1 suggesting that the presence of CD4 T cells partially inhibits CD8 T cell exhaustion. In general, it has been shown that the presence of tumor-specific CD4 T cells enhanced recruitment, proliferation, and effector functions of CD8 T cells by IFN-γ-dependent production of chemokines and IL-2 [189]. Help provided by CD4 T cells was able to further improve the functionality of CD8 T cells with high affinity TCRs [190]. On the other hand, Wong et al. reported that CD4 T cells render the tumor microenvironment permissive for infiltration by low avidity CD8 T cells [191]. Hung et al. investigated the central role of CD4 T cells in antitumor immunity and revealed that these cells not only provide signals for CD8 T cells, but have a far broader role in orchestrating immune responses to the tumor [116].

These studies demonstrate that the use of adoptive transfer of T cells in mouse tumor models is a suitable method to shed light on mechanisms of T cell regulation and is one option for successful eradication of tumors. The majority of hitherto existing models, however, utilize transgenic T cells with high affinity model antigens that do not match with the nature of most human tumors. Furthermore, this method generally skips crucial steps of establishing T cell immunity in the cancer immune cycle. In humans, the individual mutanome give rise to neoantigens of various affinities and is itself subject to a high plasticity of de novo generation and elimination of neoantigens, thereby shaping the genomic landscape. This occurs due to the genetic instability of the tumor, especially in MSI-H tumors [192]. Theoretically, generation of high affinity T cell epitopes during advanced tumor stages by a subset of cells merely slows down tumor progression, since only a fraction of the whole tumor mass bearing the mutation would induce potent responses and be eliminated. Neoepitopes that arise early in tumor development should be present in most, if not all tumor cells. Regimens targeting these neoantigens are able to induce potent remissions, as observed in the clinic today. Comparing this to mouse models using adoptive transfer of monoclonal T cells, the latter approach appears feasible to study individual aspects of T cell immunology in models of solid tumors, but it apparently fails to describe the clinically relevant course of tumor onset. The cancer immune cycle that achieves a complete remission would require checkpoint blockade such as CTLA-4- or PD-1-blocking antibodies, as well as Treg manipulation. These interventions lower the threshold of self-tolerance and trigger immune responses that may be directed to neoepitopes derived from early stages of tumor transformation.

Orchestrating immune responses by CD4 and CD8 T cells was unraveled by a study carried out by the group of Hans Schreiber, interrogating the cooperation of CD4 and CD8 T cell responses in a model of bystander killing of cancer [193]. Here, potent model antigens were expressed by a MHC-disparate tumor and the antigens were found to be displayed on cross-presenting stromal cells of tumor tissues. The potent MHC class I and II antigens elicited autologous T cell responses that led to elimination of tumor-maintaining stromal cells and subsequently induced tumor remissions. They showed that co-expression of MHC class I and II antigens was required for bystander elimination of cancer cells. The bystander elimination of cancer cells turned out to be highly efficient, since even antigen-loss variants embedded in the TME were eliminated. The inoculation of a mixture of tumor cells with separated antigens for either MHC class I or II led, in sharp contrast to double MHC class I and II-positive cancer cells, to progressive growth of mosaic tumors. The required expression of MHC class I and class II antigens by the same cell clearly demonstrated a local cooperation of CD4 and CD8 T cells during the effector phase. Moreover, experiments using mice with double positive tumors on one flank and a mixed tumor with separated class I and II antigen expression on the contralateral flank led to outgrowth of the latter tumor. These observations again demonstrate that successful bystander killing of cancer cells requires local cooperation of CD4 and CD8 T cells not only during the effector phase but also during the induction phase of tumor rejection. These results also highlight that the orchestration of immune responses by CD4 T cells is not only limited to local tumors, but even suggest that CD4 T cells are able to differentiate between individual sub-clones within the tumor tissue that are class II TSA-positive or -negative.

One feature of cancer is a unique pattern of the mutational landscape. Unlike pathogens, malignant cells are not characterized by a conserved set of immunogenic antigens. In a recent study we took advantage of this feature to establish a rodent model with tailored tumor immunogenicity to further elucidate the interplay of autologous CD4 and CD8 T lymphocytes [194]. To this end, we took advantage of transposable elements expressing oncogenic Ras that we delivered by hydrodynamic tail vein-injection to induce liver cancer in mice. Oncogenic forms of K-, H-, and NRas rapidly induced CD8 T cell responses against co-delivered model antigens, whereas no CD8 T cell responses were detectable when expression of ras was absent or when wildtype- or dominant-negative forms of ras were used. This established a direct link between an oncogene and the induction of T cell responses. Tailored tumor immunogenicity was generated by concatenated short DNA fragments coding for pre-defined single epitopes. This allowed for dissecting MHC class I and II epitopes and also for comparison of epitopes derived from immunoedited tumors with potent rejection antigens. Tumor suppression only occurred when potent MHC class I and II epitopes were both expressed by the tumor cells. Expression of antigens restricted to MHC class I epitopes induced strong CD8 T cell responses that were not capable of eliminating tumor cells without tumor-specific CD4 T cells. When MHC class II epitopes were solely expressed by the tumor, no CD4 T cell responses were detected. The results of these experiments demonstrated the mutual dependence of CD4 and CD8 T cell responses, since these cells needed to act in concert to efficiently suppress tumor development and to allow for long term survival. The autologous tumor-specific T cells showed a Th1-polarization in this study with a low, but significant cytotoxicity. However, these CD4 T cells were not able to suppress outgrowth of liver tumors without significant amounts of tumor-specific CD8 T cells. Furthermore, this study also showed the limitations of mutated CD4 and CD8 neoepitopes for cancer surveillance: although these epitopes where high affinity binders to their MHC molecule and responded well upon application of immunotherapies in corresponding parental cell lines, the host’s immune system failed to mount an immune response able to inhibit cancer outgrowth.

A similar unresponsiveness has also been described for heavily glycosylated MUC1 and Her2/neu, which was not processed by DCs and remained long term in the early endosomes [195]. In contrast, a non-glycosylated form of MUC1 was efficiently processed by DCs. These results suggest that TSAs may often be inappropriate T cell targets for the immune system and require therapeutic interventions or modifications to overcome tolerance. The interplay of CD4 and CD8 T cell responses may also counteract tolerance induction by the tumor and lower the threshold of tumor immune recognition for CD8 T cell epitopes by induction of epitope spreading to antigens that do not trigger tumor remission without CD4 help. Surman et al. showed that adoptive transfer of Th1-polarized CD4 T cells induced tumor-specific CD8 T cell responses and tumor remission [196]. The authors suggested that lowering the threshold for immune recognition was achieved by CD4 T cells that enhanced the ability of APCs to trigger CTLs to a model antigen. Investigating the interactions between CD4 T cells with APCs revealed CD40-CD40L crosstalk to trigger effective CTLs in a CCL5-dependent manner [197]. In the same study, CCR5-positive DCs were attracted to the tumor site and were then licensed by CD4 T cells prior to the generation of CD8 T cell immunity. This CD4-mediated CD40-dependent licensing of DCs as a precondition for functional CTLs has also been shown in vitro for human cancers [198]. Another study demonstrating the importance of CD4 T cells in a murine model showed that CD8 T cell tolerance to the self-antigen MDM-2 could be overcome by adoptive transfer of TCR-modified CD4 T cells [199]. TCR-engineered CD8 T cells displayed an exhausted phenotype lacking cytotoxic function. Engineered CD4 T cells allowed for T cell help that facilitated a partial reversal of tolerance with the same MDM-2-specific TCR. Similar results of converting tolerized CD8 T cells were observed in a rodent TCR transgenic model with weakly immunogenic tumors [200]. Although transgenic CD4 and CD8 T cells were transferred and CD8 T cells were able to recognize their corresponding cross-presented antigen in draining lymph nodes, MHC class II antigen presentation on CD8-positive dendritic cells and subsequent priming of CD4 T cells was defective due to the influence of the TME. In accordance with studies mentioned above, in which CD8 T cell immunity was triggered or reactivated by CD4 lymphocytes, this work revealed that T cell immunity entirely failed to counteract tumor growth when the generation of CD4 T cell responses was interrupted.

On the one hand, these studies show the importance of T cell responses that may often dictate tumor regression or progress. On the other hand, CD4 and CD8 T cell responses frequently fail to maintain proper function during tumor remissions. Therapeutic intervention to sustain and promote T cell immune reactions directed to the tumor is therefore a major aim in clinical oncology. The rational design of such therapies will require a detailed understanding of CD4 and CD8 T cell regulation including their immune checkpoint signaling.

## Interplay of CD4 and CD8 T cells in cancer immunotherapies

Cancer immunotherapies are designed to activate or reactivate a therapeutic antitumor activity in the immune system. There are different strategies to modulate the immune system and to induce or expand tumor-specific T cells: administration of therapeutic cancer vaccines against tumor-associated antigens [201–203]; application of cytokines, such as IL-2 [204]; cell based therapies including adoptive cell transfer of naturally occurring tumor-reactive lymphocytes [205] or genetically modified autologous T cells expressing chimeric antigen receptors (CARs) such as CD19 [206]; the use of oncolytic virotherapy to mediate tumor cell death and stimulate T cell-mediated tumor immunity [97, 207–209]; and immune checkpoint blockade targeting T cell regulatory pathways [210]. Albeit most pharmaceuticals are used off-the-shelf, the latter approach provides an attractive treatment option, resulting from the therapeutic effect on the immune system that stimulates T cell responses and subsequently target predominantly individual neoantigens of the tumor [211, 212].

In this regard, cancer mouse models in combination with next generation sequencing of murine cell lines were helpful to rapidly identify potent T cell epitopes that were of relevance for T-cell-mediated tumor clearance upon various immunotherapeutic regimens as described above. Valuable knowledge about molecular mechanisms of checkpoint blockade and regulation of the immune system arose from these models. However, with regard to checkpoint blockade, a critical differentiation between animal models and clinical studies in corresponding tumor entities appear reasonable. Whereas the neoantigenome of human cancers in individual patients are unique and within a certain range concerning accumulated number of mutations [213], mouse models are often based on well described cell lines with little variation and are, as such, biased. Also the method of tumor induction to generate cell lines is of vital importance here. The use of carcinogenic agents leads to abundant numbers of mutations that greatly exceed the mutation rates of comparable tumor types in humans. Compared to chemically-induced tumors, established tumor cell lines from genetically modified mouse models show a much lower mutation rate and may therefore be more suitable to investigate checkpoint blockade in rodents [75]. Additionally, there is a significant discrepancy between high affinity neoepitope occurrence in established mouse models and the frequency of high affinity antigens in the majority of patients. This is reflected by the overall response rate upon immune checkpoint blockade and the immune profiling data of van Rooij et al. that revealed a single ipilimumab-responsive MHC class I-restricted neoepitope candidate among 1075 nonsynonymous single nucleotide variants in a patient with melanoma [27]. Whether in vivo results from mouse models derived from a given cell line can be generalized to the corresponding tumor entity in humans remain, as mentioned above, questionable.

In 2011, the FDA approved ipilimumab, the first antibody targeting CTLA-4, for the treatment of metastatic melanoma [214]. Thus, clinical application required several years of research and development until the first evidence for antitumor immunity was established [215]. Since approval of ipilimumab, immune checkpoint therapies have established their position among the most effective cancer treatments available for patients today [216]. Unfortunately, despite of the high number of already approved drugs, immune-related adverse events are a significant problem. In the case of ipilimumab, a positive response is observable in one-fifth of the patients [217]. However, 10–35% of recipients also suffer from severe side effects [218–220]. In general, immune related adverse events of checkpoint inhibitors also depend on the target molecule. Compared to ipilimumab, the PD-1 checkpoint inhibitor pembrolizumab showed less high-grade toxicity in a large clinical study [221]. Until now, PD-1 checkpoint inhibitors such as nivolumab and pembrolizumab are regarded as the most effective immunotherapies with the best safety profile [222]. The majority of immune related adverse events are, if diagnosed timely, completely reversible upon proper management [223]. Compared to treatment of lung cancer with conventional regimens such as chemotherapy or radiation, immunotherapies generally are associated with less side effects and improve daily living [224]. Without much doubt similar results concerning this issue will be evident upon comparison of immunotherapy with conventional therapies in other tumor entities.

Indeed, melanoma patients with PD-L1 positive tumors that received a combination of nivolumab and ipilimumab showed the same progression-free survival as the patients in the nivolumab group [225]. In contrast, patients with PD-L1-negative tumors had longer progression-free survival in the combination therapy group, compared to nivolumab or ipilimumab alone. This shows the importance of the PD-L1 status of tumors as a biomarker for efficacy of immunotherapies, T cell responses and frequency of tumor remissions during checkpoint blockade. Although therapeutic interventions in cases of advanced melanoma and recurrent small-cell lung cancer (SCLC) using a combination of nivolumab and ipilimumab induces a considerably higher response rate compared to corresponding monotherapies, this combination also led to an increased higher number of treatment-related adverse events [182, 225, 226]. A phase 3 trial of patients with unresectable or metastatic melanoma who progressed after ipilimumab treatment demonstrated that some patients benefit from PD-1 checkpoint blockade using nivolumab [227]. Patients in this trial showed a better response rate after nivolumab treatment (31.7%), compared to patients treated with chemotherapy (10.6%). The reason why some patients responded to nivolumab again, while others were resistant to this immunotherapy remains obscure. However, these results demonstrate that acquired resistance to an immune checkpoint therapeutic is restricted to the target molecule and allows for effective continuation of immunotherapeutic treatment with other checkpoint targets. Thus, diverse immunological checkpoints enable continued adaptive immune response to cancer, which is mediated by cytotoxic T cells. Patients that received nivolumab had fewer treatment-related adverse events, compared to chemotherapy-treated patients. Weber et al. confirmed that patients with PD-L1-positive tumors also had a markedly better objective response (43.6%) compared to patients with PD-L1 negative tumors (20.3%).

There are a handful of studies that investigate T cell responses in recipients, providing valuable information on the human immune reactions. Such evidence suggests that CTLA-4 treatment induces a CD8 T cell response against new targets in melanoma patients, rather than boosting a pre-existing immune response [172]. The exact mechanism by which the T cells of patients during immune checkpoint blockade are triggered is yet to be elucidated. Initially, it was suggested that upon binding CTLA-4 the monoclonal antibody blocks its inhibitory effect on both effector and regulatory T cells, thereby increasing effector T cell activity sufficient for induction of tumor regression. Simpson et al. studied this mechanism and refined this view in a mouse model of cancer [228]. First, they showed that CD4 T cells are crucial for tumor rejection. Mice lacking CD4 T cells were unresponsive to CTLA-4 antibody treatment when challenged with a transplantable melanoma cell line. CTLA-4 treatment has been documented to enhance the ratio of intratumoral Teff and Tregs of the CD4 T cell compartment [112, 229, 230], an effect that was proposed to be important for the success of immunotherapy [231]. To elucidate the mechanisms responsible for this effect, the authors of this study injected Trp1-specific CD4 T cells into mice that were challenged with a melanoma cell line, and showed that CTLA-4 inhibition increased both Trp1-specific Teff and Treg cell numbers, while reducing intratumoral accumulation of Trp1-specific Tregs rapidly by depletion. After establishing that these results are neither observable in complement deficient nor in γ-chain knockout mice, they identified antibody-dependent cell-mediated cytotoxicity (ADCC) as the driving mechanism of tumor-residing Treg depletion, mediated by Fcγ receptors, mainly FcγRIV. They could also show that FcγRIV-positive macrophages facilitating Treg depletion were enriched in the microenvironment of tumors in mice responding to CTLA-4 treatment, underlining once again the importance of modifying the tumor microenvironment for a successful immune checkpoint blockade therapy.

The antitumor activity of CTLA-4 therapy is also strongly dependent on the isotype. Applications of identical CTLA-4 antibodies with diverse isotypes have shown profound differences in their efficacy and features to stimulate immunity throughout different mouse strains and MHC haplotypes [232]. In contrast to other isotypes, only IgG2a was highly effective in eradication of established tumors. It is important to note that antitumor activity of CTLA-4 IgG1 was comparable to the isotype control, stressing the importance of the isotype of monoclonal antibodies for therapeutic outcomes. Selby et al. also demonstrated the selective depletion of Tregs from the tumor site by CTLA-4-IgG2a and reported significant improvement of the Teff to Treg ratio. Interestingly, Tregs were expanded by all CTLA-4 isotypes at peripheral sites. However, constitutive expression of CTLA-4 was found only on tumor Tregs, providing a rationale for selective depletion of this cell type from tumors by ADCC or antibody-dependent cellular phagocytosis. It has to be noted that most human Fcγ receptors have the same CD and name as mouse Fcγ receptors. Their function in binding IgGs and their expression pattern is, however, quite different [233]. For instance human IgG1 binds to all activating Fcγ receptors, whereas mouse IgG1 binds only to activating FcγRIII. Hence, Fc receptor–immunoglobulin interactions and their subsequent effector signaling vary throughout different species and have to be considered accordingly when monoclonal antibodies for immunotherapeutic applications are developed and optimized. In clinical applications, examples for CTLA-4 antibodies are tremelimumab with an IgG2 isotype and ipilimumab with an IgG1 isotype. IgG2 in tremelimumab was chosen to minimize possible detrimental effects of cytotoxicity on activated T cells and cytokine release syndrome [234]. Moreover, tremelimumab did not affect Treg frequency. It instead increased the frequency of IL-2-secreting CD4 T cells and IFN-γ-secreting CD4 and CD8 T cells [235]. Hence, tremelimumab induces tumor remissions mainly by direct activation of Teff cells in responding patients. In case of ipilimumab the IgG1 isotype is capable of activating non-classical macrophages by binding to CD16 that leads to a selective depletion of Tregs in vitro [234]. Finally, these studies also demonstrate significant differences in monoclonal antibody-mediated effects determined by the isotype for cancer therapies in the human system. Whether patients can benefit from a combination of tumor evaluation and subsequent isotype selection of therapeutic antibodies remains to be determined.

The awareness that isotypes can have a crucial influence on therapeutic outcomes led to isotype engineering and optimization that has been performed on a CD25-depleting monoclonal antibody to increase responsiveness to PD-1 therapy by exploiting the mechanism of ADCC to deplete regulatory T cells [236]. Combining Fc-optimized anti-CD25 and PD-1 antibodies enhanced proliferation of effector T cells, CD4 and CD8 alike, and improved their IFN-γ production. Moreover, this markedly improved the CD8/Treg ratio in the tumor tissue in favor of tumor-infiltrating CD8 T cells. This combination led to efficacious tumor eradication of mouse cancer cells that were resistant against PD-1 checkpoint blockade alone. These findings clearly highlight the importance of immunotherapies that address several types of immune cells, thus preventing the emergence of cancer resistances and improving the responsiveness to treatment.

The PD-1-blocking antibody pembrolizumab has been shown to elicit an increased proliferation of both, Foxp3-negative and Foxp3-positive CD4 as well as CD8 T cells in stage IV melanoma patients, displaying the most significant effects in the PD-1 expressing cells of these populations [237]. Interestingly, while 74% of patients in this study displayed an increase in PD-1-positive CD8 T cell proliferation following PD-1 checkpoint blockade, a clinical response was apparent in only 38% of the patients. After immune profiling of peripheral blood samples, a population of circulating Tex cells could be identified as a major target of PD-1 blockade, showing a peak of increase in Tex cell proliferation or reinvigoration, generally about 3 weeks after starting an immunotherapeutic regimen. These cells contained T cell clones identical to tumor-infiltrating T cells. Antigen burden was estimated based on tumor lesion-size, and was found to correlate with Tex cell reinvigoration before and after PD-1 treatment. Consistent with the results of the aforementioned study, Kamphorst et al. have shown that following PD-1 targeted therapy in non-small cell lung cancer (NSCLC) patients, their CD4 and CD8 T cells also show an increase in Ki67 expression [238]. PD-1-positive CD8 T cells were again identified to be the most responsive immune cell subset, with a proliferative activation in 70% of the patients. Upon further analysis of these cells, they proved to have an effector-like phenotype and co-express CTLA-4 with PD-1 as well as the costimulatory molecules CD27, CD28, and inducible co-stimulator (ICOS). This immunological response translated into a clinical effect in 80% of the patients. The authors suggested that the Ki67-positive PD-1-positive CD8 T cells have mostly tumor-specific TCRs. Lack of knowledge about tumor-specific T cells made it difficult to prove this claim, but they showed that PD-1 targeted therapy does not change the proliferation rate of EBV-specific PD-1-positive CD8 T cells.

Nevertheless, both considering the immune regulatory factors that prevent the development of T cell responses and using appropriate markers that impact the efficacy of immunotherapy can directly translate to higher response rates of cancer patients, as demonstrated in a recent study of NSCLC [239].

To sum up, immunotherapies activate both CD4 and CD8 T cells, albeit to a different extent. Depending on their interplay and the combination of applied therapies, this effect can be sufficient to produce a positive clinical outcome, providing a valuable alternative to conventional cancer treatment options.

## Conclusions

As we have seen from the literature, a malignant disease overcomes several tumor-suppressive mechanisms of the host by a plethora of adaptations. As such cancer is an ever evolving disease that also includes escape from counter-regulations of the immune system. In advanced stages of cancer, when the disease is usually detected, CD4 and CD8 T cell responses are often ineffective and at best only slow down tumor progression due to low tumor immunogenicity, influence of the TME, or an interruption of the cancer immune cycle. Consequently, the central aim in clinical oncology is to reactivate T cell responses or, in other words, to convert a ‘cold tumor’ into a ‘hot’ one. This colloquial term covers overall issues of checkpoint blockade for cancer therapy including unpredictability of treatment responses in individual patients and low response rates in general, developments of therapeutic resistance, and the need to establish additional supportive regimens to maintain or increase tumor immunogenicity during immunotherapy. Hence, development of novel approaches would not be based on monotherapies, but rather combine strategies that help to overcome any therapy-induced acquired resistances of the tumor in order to enable a continued therapeutic success.

Development of novel treatment approaches would include the use of checkpoint inhibitor combinations, or combinations with other available clinical drugs that demonstrate the best manageable safety profile and synergize with immunotherapy.

Establishing these kinds of approaches can only be successful upon a thorough understanding of molecular mechanisms of the immune system, in particular the detailed understanding of CD4 and CD8 T lymphocyte responses against tumors. Thus, characterization and investigation of the interplay of these responses should be performed in parallel with clinical trials for the rapid development of highly effective immunotherapies for the treatment of various types of cancers.

## Abbreviations

ADCC: Antibody-dependent cell-mediated cytotoxicity
AML: Acute myeloid leukemia
APC: Antigen-presenting cell
CAR: Chimeric antigen receptor
CD: Cluster of differentiation
CIITA: MHC class II transactivator
CDK4: Cyclin-dependent kinase 4
CRC: Colorectal cancer
CTL: Cytotoxic T lymphocyte
CTLA-4: Cytotoxic T lymphocyte-associated protein 4
DC: Dendritic cell
EBV: Epstein–Barr virus
ESCC: Esophageal squamous cell carcinoma
Fc: Fragment, crystallizable
FcγR: Fc gamma receptor
Foxp3: Forkhead box P3
*Gzmb*: *Granzyme B gene*
Her2/neu: Human epidermal growth factor receptor 2
HNSCC: Head and neck squamous cell carcinoma
HRas: Harvey rat sarcoma
ICOS: Inducible costimulator
IFNAR1: Interferon-alpha/beta receptor alpha chain
IFN-γ: Interferon gamma
IL: Interleukin
KRas: Kirsten rat sarcoma
LAG-3: Lymphocyte-activation gene-3
LCMV: Lymphocytic choriomeningitis virus
MHC: Major histocompatibility complex
NSCLC: Non-small cell lung cancer
NK cell: Natural killer cell
NKT cell: Natural killer T cell
NRas: Neuroblastoma rat sarcoma
NY-ESO-1: New York esophageal squamous cell carcinoma-1
p16^INK4a^: Cyclin-dependent kinase inhibitor 2A
PD-1: Programmed death-1
PDA: Pancreatic ductal adenocarcinoma
PD-L1: Programmed death ligand-1
*Prf1*: *Perforin1 gene*
PTEN: Phospatase and tensin homolog
RAG2: Recombination activating gene 2
RORγt: Retinoic acid-related orphan receptor gamma t
SASP: Senescence associated secretory phenotype
SCLC: Small-cell lung cancer
TAA: Tumor-associated antigen
TCR: T cell receptor
Teff: Effector T cell
Tex: Exhausted CD8 T cell
TGF-β: Transforming growth factor beta
Th0/1/2/17: T helper cell commitment 0/1/2/17
TIGIT: T cell immunoreceptor with Ig and ITIM domains
TIM-3: T cell immunoglobulin and mucin domain containing protein 3
TME: Tumor microenvironment
Tmem: Memory T cell
TNF: Tumor necrosis factor
Treg: Regulatory T cell
TRP1: Tyrosinase-related protein 1
TSA: Tumor-specific antigen
